# Supplementary material for: mzQuality: An Open-Source Software Tool for Quality Monitoring and Reporting of Targeted Mass Spectrometry Measurements
Source: J Am Soc Mass Spectrom. 2025 Jul 25;36(8):1669–76. doi: 10.1021/jasms.5c00073 (PMC12333366; doi:10.1021/jasms.5c00073)
Supplement: Supplementary file 1 [file js5c00073_si_001.pdf]

Supporting Information:

mzQuality: An open-source software tool for quality monitoring and reporting of targeted mass spectrometry measurements

Marielle van der Peet[1], Pascal Maas[1], Agnieszka Wegrzyn[1], Lieke Lamont[1], Ronan Fleming[1,2], Constance Bordes[3], Stéphanie Debette[3,4,5], Amy Harms[1], Thomas Hankemeier[1], Alida Kindt*[1]

[1] Metabolomics and Analytics Centre, Leiden Academic Centre for Drug Research, Leiden University, Einsteinweg 55, Leiden, The Netherlands
[2] Digital Metabolic Twin Centre, School of Medicine, University of Galway, University Rd, Galway, H91 TK33, Ireland
[3] University of Bordeaux, INSERM, Bordeaux Population Health Research Center, UMR1219, Bordeaux, F-33000, France
[4] Department of Neurology, Institute for Neurodegenerative Diseases, Bordeaux University Hospital, Bordeaux, F-33000, France
[5]Institut du Cerveau (ICM), Paris Brain Institute, INSERM U1127, UMR CNRS 7225 Paris, Sorbonne Université, Assistance Publique des Hôpitaux de Paris, Paris 75013, France

***Address correspondence to:**
Alida Kindt, Einsteinweg 55, 2333 CC, Leiden, The Netherlands, +31715272727, a.s.d.kindt@lacdr.leidenuniv.nl

Supporting tables

Table S1. Batch design, for example data; see SI_002.xlsx

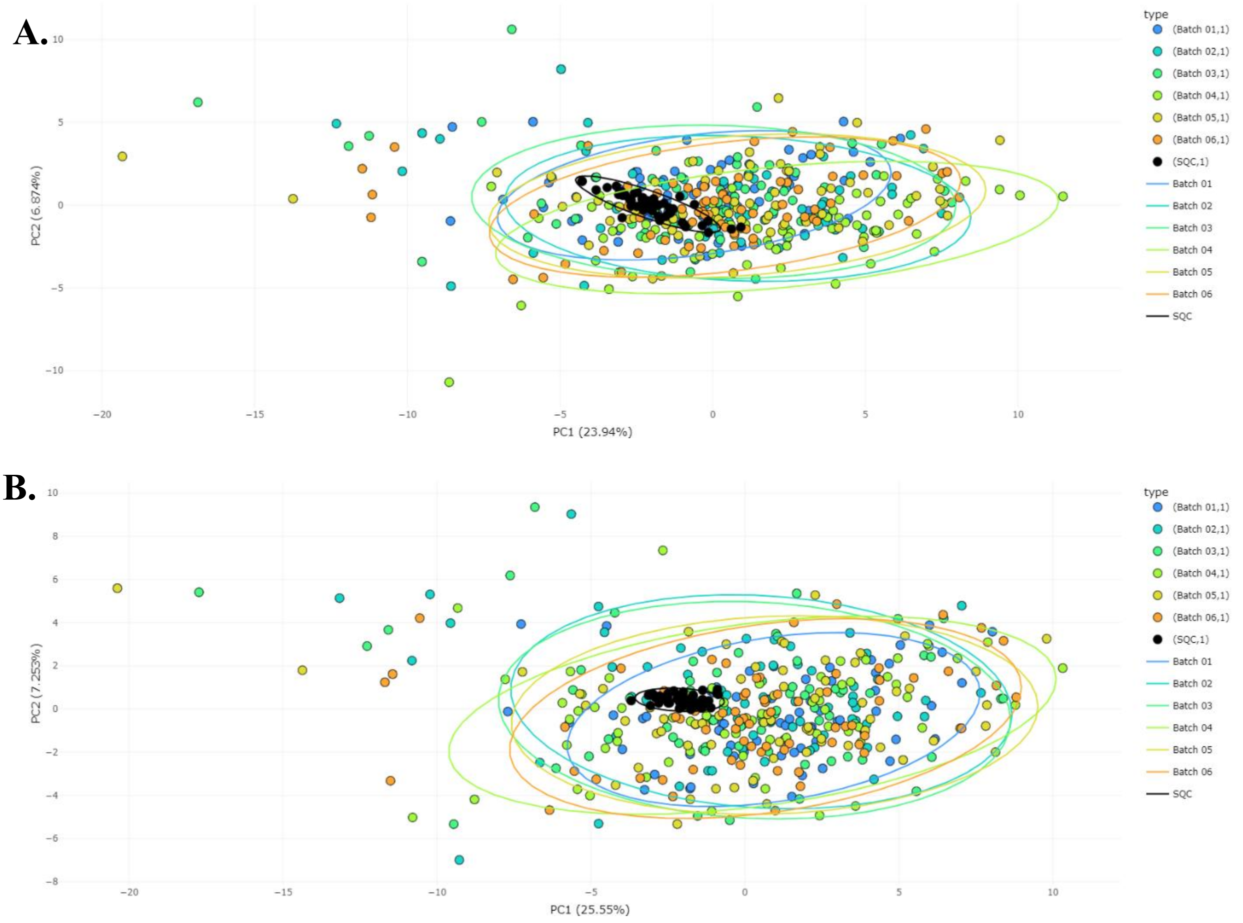

Figure S1: PCA plot before and after between-batch correction. **A**. PCA plot of batch 1-6 before correction. **B**. PCA plot of batch 1-6 after correction. The samples are grouped by batch and show a 95% confidence interval. After batch correction, the SQC samples are more aligned in the middle of the two batches.

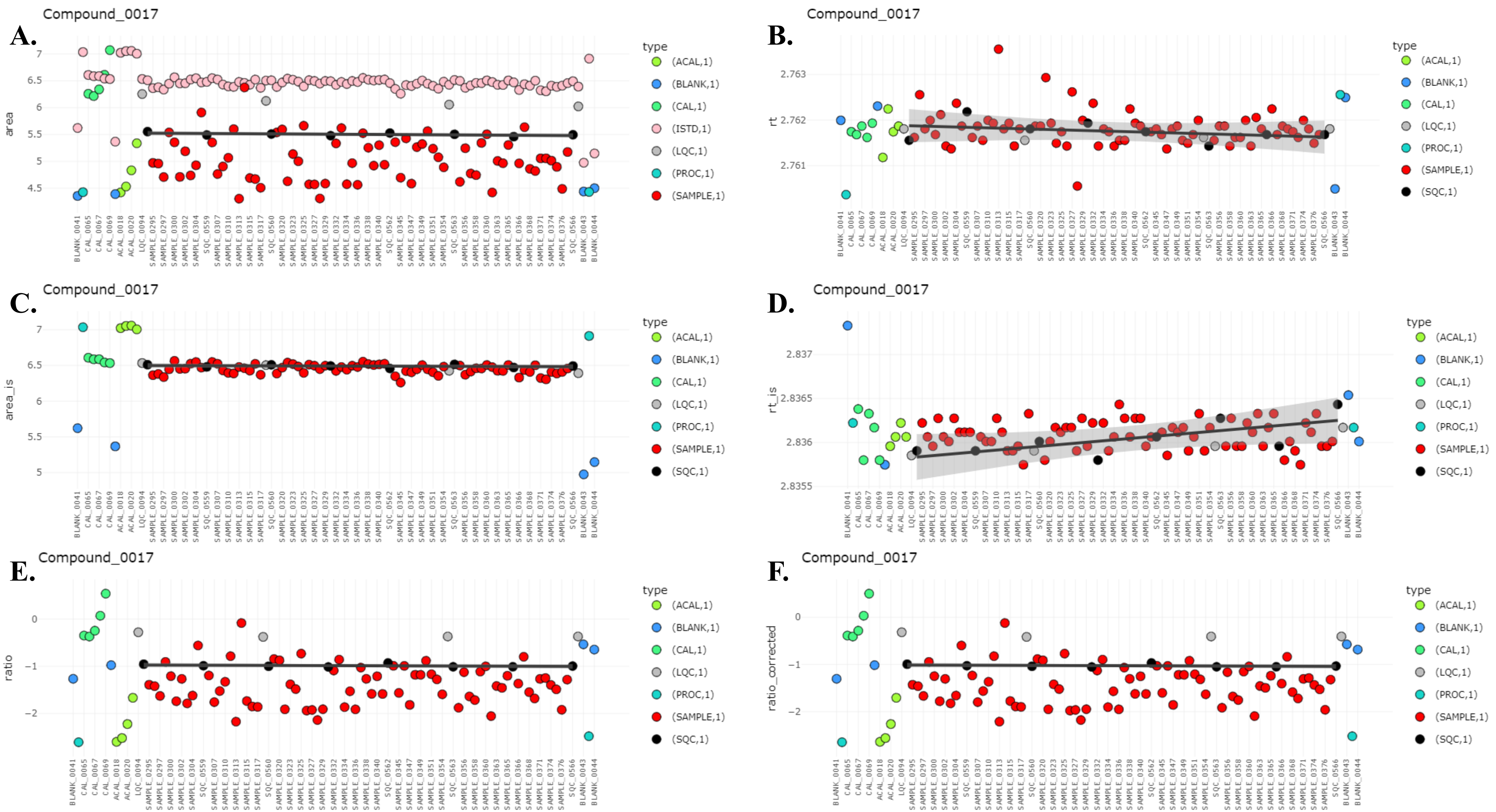

Figure S2: Individual plots of compound 17 in batch 5 including all sample types. The y-axis displays the log2 transformed corrected ratios, and the x-axis displays the injection number. **A**. Area including the internal standard area. **B.** Retention time **C**. Area of the corresponding internal standard **D**. Retention time of the internal standard (in minutes). **E**. Ratio of the compound area to the internal standard area (area/area_is). **F.** Corrected ratio after between-batch correction.
